# Supplementary material for: MetaboT: an LLM-based multi-agent framework for interactive analysis of mass spectrometry metabolomics knowledge graphs
Source: J Cheminform. 2026 Jul 24;18:146. doi: 10.1186/s13321-026-01257-8 (PMC13689178; doi:10.1186/s13321-026-01257-8)
Supplement: Supplementary file 1 [file 13321_2026_1257_MOESM1_ESM.pdf]

Additional file 1: Supplementary Methods and Results

MetaboT: An LLM-based Multi-Agent Framework for Interactive Analysis of Mass Spectrometry Metabolomics Knowledge Graphs

Journal of Cheminformatics — Software article

Contents

S1 Extended Framework Description 2
- S1.1 LangChain and LangGraph . . . 2
- S1.2 Formal Agent Definition . . . 2
- S1.3 System Architecture Overview . . . 2

S2 Per-Agent Detailed Descriptions 3
- S2.1 Entry Agent . . . 3
- S2.2 Validator Agent . . . 4
- S2.3 Supervisor Agent . . . 4
- S2.4 KG Agent (Knowledge Graph Agent) . . . 4
- S2.5 SPARQL Query Runner Agent . . . 4
- S2.6 Interpreter Agent . . . 5

S3 Per-Tool Detailed Descriptions 5
- S3.1 PlantDatabaseChecker . . . 5
- S3.2 ChemicalResolver . . . 5
- S3.3 SMILESResolver . . . 6
- S3.4 TargetResolver . . . 6
- S3.5 TaxonResolver . . . 6
- S3.6 FileAnalyzer . . . 6
- S3.7 GraphSparqlQAChain . . . 7
- S3.8 WikidataStructureSearch . . . 7
- S3.9 OutputMerger . . . 7
- S3.10 Interpreter (tool) . . . 8
- S3.11 SpectrumPlotter . . . 8

S4 SPARQL Query Generation and Refinement — Extended Description 8

S5 Agent Interaction — Extended Description 9
- S5.1 LangSmith Monitoring . . . 11

S6 Detailed Evaluation Protocol 11
- S6.1 Automated Evaluation Framework . . . 11
- S6.2 Iterative Evaluation Rounds . . . 11
- S6.3 Manual Evaluation . . . 11
- S6.4 Evaluation Scope . . . 11
- S6.5 Benchmark Dataset Construction . . . 11

S7 Hardware and Software Requirements 12

S8 Full Evaluation Results 12

S1 Extended Framework Description

S1.1 LangChain and LangGraph

MetaboT is built on LangChain (version 0.3.27) [1] and LangGraph (version 0.3.34) [2]. LangChain provides a flexible framework for developing LLM-based applications and organises its abstractions into four principal component types:

- **LLM.** The language model that underpins each agent's core reasoning, including translating natural-language queries into SPARQL.
- **Prompts.** Structured instructions that guide the LLM towards targeted outcomes; in MetaboT, each agent has a dedicated system prompt that encodes domain rules, schema examples, and task-specific few-shot demonstrations.
- **Chains.** Compositions of prompts and LLMs that produce coherent, multi-step outputs; the `GraphSparqlQAChain` tool is the primary chain used for SPARQL generation and refinement.
- **Agents.** Units that combine an LLM chain with one or more tools, where the LLM acts as a reasoning engine to select and invoke appropriate tools.

LangGraph builds on LangChain by structuring the workflow as a *directed state graph*: each node represents a discrete computational step (i.e., an agent or tool), and each edge represents message-passing between nodes. The graph maintains a mutable shared state that evolves as each agent completes its task, enabling adaptive routing and context propagation throughout the query-processing pipeline.

S1.2 Formal Agent Definition

Each agent A_i in MetaboT's workflow is formalised as the tuple [3]:

$$A_i = (L_i,\ P_i,\ T_i) \tag{S1}$$

where:

- L_i is the language model that guides the agent's decision-making (GPT-4o for the primary configuration; GPT-4o mini for the comparative evaluation).
- P_i is the prompt customised for the agent's specific purpose, encoding domain knowledge, schema examples, and task constraints.
- T_i is the set of functions or tools the agent may invoke to fulfil its task.

S1.3 System Architecture Overview

Figure S1 shows the comprehensive structure of MetaboT's multi-agent framework. Purple nodes denote the six core agents (Entry, Validator, Supervisor, Knowledge Graph, SPARQL Query Runner, Interpreter), green nodes represent the eleven supporting tools (`PlantDatabaseChecker`, `ChemicalResolver`, `SMILESResolver`, `TargetResolver`, `TaxonResolver`, `FileAnalyzer`, `GraphSparqlQAChain`, `WikidataStructureSearch`, `OutputMerger`, `Interpreter`, `SpectrumPlotter`), and light-blue nodes indicate external resources (ENPKG SPARQL endpoint, GNPS API, ChEMBL API, Wikidata endpoint). A dashed *New Agent* node from the Supervisor illustrates the modular expansion capability of the graph architecture.

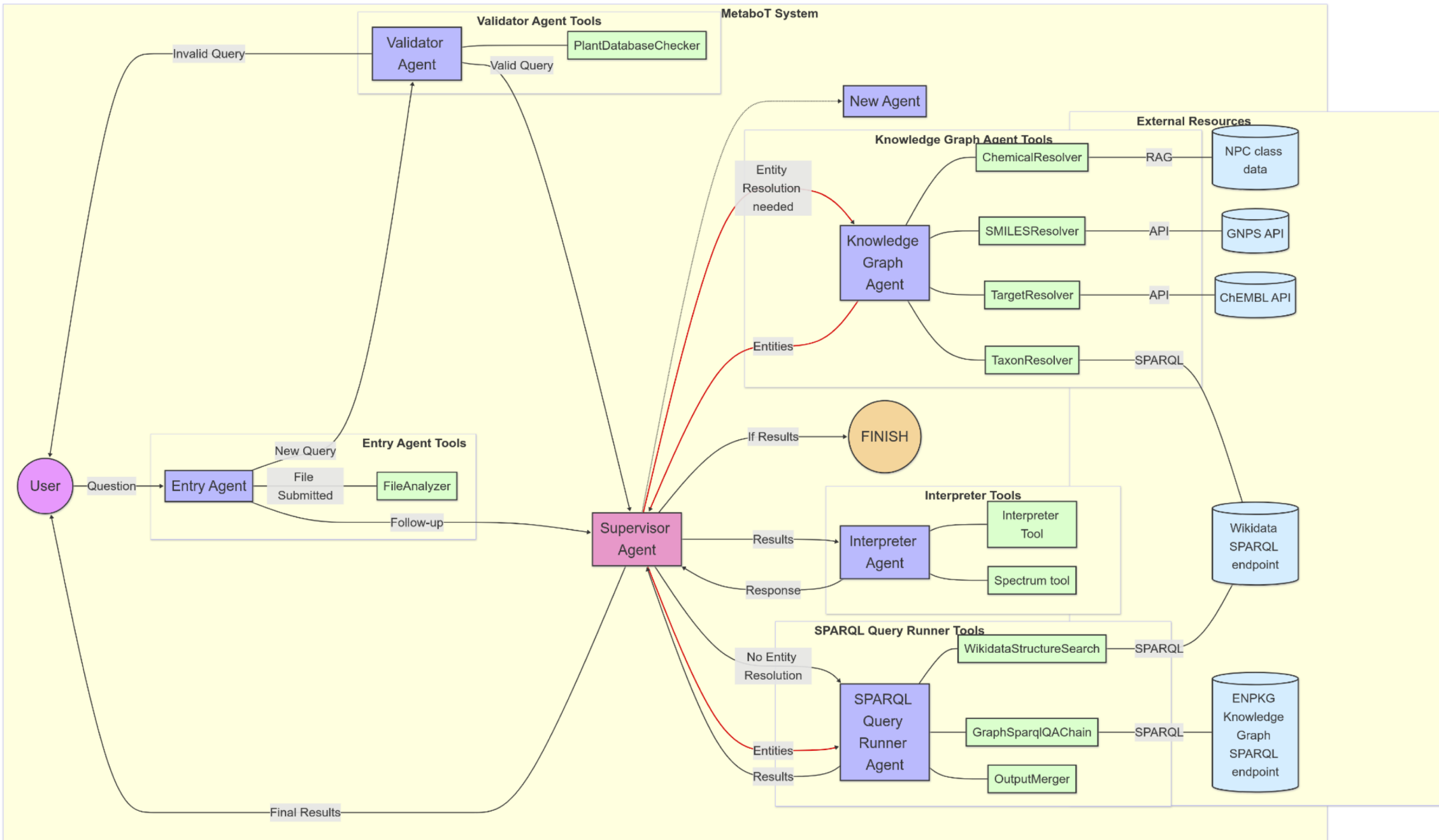

Figure S1: **MetaboT system architecture.** Comprehensive diagram of the multi-agent framework. Purple nodes: core agents; green nodes: supporting tools; light-blue nodes: external resources. Directional arrows trace information flow from the user's natural-language query through the processing pipeline to the final SPARQL-derived response. The dashed *New Agent* node illustrates the modular extension capability.

S2 Per-Agent Detailed Descriptions

The main text summarises the role of each agent. This section provides the full description of each agent's key functions and processing approach as implemented in MetaboT v1.0.

S2.1 Entry Agent

Purpose. Acts as the gateway to MetaboT, determining whether a query is a new question or a follow-up to prior interactions.

Key functions.

- Processes user-provided files via the `FileAnalyzer` tool.
- Forwards new queries to the Validator Agent.
- Retrieves context for follow-up questions from conversation history.

Processing approach. The agent classifies queries into two categories:

1. *New Knowledge Questions* — queries that require new information from the knowledge graph, forwarded to the Validator Agent for scope checking.
2. *Help Me Understand Questions* — follow-up queries seeking clarification on previous answers, routed directly to the Supervisor Agent using stored conversation context, thereby avoiding redundant pipeline invocations.

S2.2 Validator Agent

Purpose. Ensures that user questions are appropriate for querying the knowledge graph before further processing.

Key functions.

- Confirms query validity and alignment with the schema and available data.
- Uses the `PlantDatabaseChecker` tool to verify whether plant or taxon names mentioned in the query are present in the ENPKG.
- Routes valid questions to the Supervisor Agent; returns specific feedback for invalid ones.

Processing approach. The agent employs in-context learning [4] and few-shot examples by embedding the knowledge-graph schema directly in its prompt. This enables cross-checking against the knowledge graph's capabilities and ensures schema compliance through comparison with labelled examples of valid and invalid questions.

S2.3 Supervisor Agent

Purpose. Acts as the central coordinator, routing user queries to appropriate agents based on content analysis.

Key functions.

- Identifies key entities in the query (taxon names, chemical names, biological targets, *etc.*).
- Delegates entity-resolution tasks to the KG Agent when required.
- Passes processed outputs to the SPARQL Query Runner Agent.

Processing approach. The agent follows explicit guidelines for task delegation, entity-specific processing, and multi-step query workflows. Its system prompt includes detailed worked examples demonstrating proper task routing and error-handling procedures, enabling adaptive processing based on query content.

S2.4 KG Agent (Knowledge Graph Agent)

Purpose. Resolves entities mentioned in user queries to precise, authoritative identifiers for use in SPARQL query construction.

Key functions. Employs specialised tools to retrieve standardised identifiers:

- `ChemicalResolver` for chemical class names (retrieval-augmented generation on a reference file).
- `SMILESResolver` for molecular structures (GNPS API [5]).
- `TargetResolver` for biological targets (ChEMBL API [6]).
- `TaxonResolver` for taxonomic entities (SPARQL on the Wikidata endpoint [7]).

Processing approach. By grounding each entity in external APIs, retrieval-augmented generation, or live SPARQL queries rather than relying on the LLM's parametric knowledge, the KG Agent substantially reduces hallucinated identifiers in downstream query construction.

S2.5 SPARQL Query Runner Agent

Purpose. Facilitates SPARQL query generation and data retrieval from the knowledge graph.

Key functions.

- *Input preparation*: if the question requires cross-referencing with Wikidata compound annotations, the agent separates the Wikidata sub-question and routes only the non-Wikidata component to `GraphSparqlQAChain`, calling `WikidataStructureSearch` in parallel; otherwise, the full question with resolved entities is sent directly to `GraphSparqlQAChain`.
- Ensures that the user's question, resolved entity identifiers, and schema details are properly structured before query generation.
- Manages query execution and result formatting.
- Uses the `OutputMerger` tool to combine results from the ENPKG endpoint and the Wikidata endpoint when both are consulted.

Processing approach. The agent emphasises structured input preparation over direct query generation, ensuring precision through comprehensive context provision. It handles external data sources when necessary and formats results appropriately for downstream processing by the Interpreter Agent.

S2.6 Interpreter Agent

Purpose. Bridges the gap between raw SPARQL query results and user-friendly insights.

Key functions.

- Analyses outputs from the SPARQL Query Runner Agent or user-submitted files.
- Produces clear textual summaries of query results.
- Generates visualisations (bar charts, mass spectrum plots, network diagrams) when requested.

Processing approach. Depending on the request type, the agent provides structured inputs to its tools, which analyse files and generate visualisations. For data visualisation, it dynamically generates and executes Python code within a secure, custom-built local subprocess sandbox to produce JSON code for Plotly [8] graphs. The agent follows detailed guidelines to ensure all data are presented in an accessible, informative format tailored to the user's query.

S3 Per-Tool Detailed Descriptions

S3.1 PlantDatabaseChecker

Objective. Verify the presence of plant names within a pre-existing database to filter queries based on data availability.

Input. Plant name string extracted from the user query.

Process. Loads a CSV file containing the plant and taxon names present in the ENPKG analysis and searches for exact string matches in the designated column.

Output. Returns confirmation of the plant's presence or absence, with appropriate error handling for file-access issues.

S3.2 ChemicalResolver

Objective. Resolve chemical class names to standardised NPCClass URIs for incorporation into SPARQL queries.

Input. Chemical class name string extracted from the user query.

Process. Matches chemical names to NPCClass URIs using a Facebook AI Similarity Search (FAISS) vector database [9] with OpenAI embeddings [10]. A precompiled CSV file extracted from the knowledge graph provides the NPCClass reference data; this is processed into searchable chunks, converted to embedding vectors, and stored in a FAISS retriever object.

Output. Returns the closest NPCClass URI with associated type information.

S3.3 SMILESResolver

Objective. Convert SMILES representations [11] of molecular structures to standardised InChIKeys [12] for chemical interoperability.

Input. SMILES string representing the molecular structure of interest.

Process. Submits the SMILES string to the GNPS API [5] to retrieve the corresponding InChIKey.

Output. Returns the standardised InChIKey with process-status logging.

Error handling. Implements robust logging for API request failures and exception handling with system notification.

S3.4 TargetResolver

Objective. Map biological target names to standardised ChEMBL Target IRIs [6].

Input. Biological target name string.

Process. Queries the ChEMBL REST API for matches based on the target name and parses XML responses to extract and format ChEMBL target IDs as IRIs.

Output. Returns the formatted ChEMBL Target IRI.

Error handling. Logs both successful and unsuccessful API calls and provides suggestions for name refinement when no match is found.

S3.5 TaxonResolver

Objective. Resolve taxonomic names to their corresponding Wikidata IRIs [7].

Input. Taxonomic name string (genus, species, or higher taxon).

Process. Constructs and executes a SPARQL query on the Wikidata endpoint to retrieve the unique IRI associated with the taxon name.

Output. Returns the Wikidata IRI for the taxonomic entity.

Error management. Implements robust error handling for unexpected response formats and connectivity issues, with clear outcome logging.

S3.6 FileAnalyzer

Objective. Analyse user-provided files and generate content summaries for downstream processing.

Input. User-uploaded files and a session identifier.

Process. Initialises session-specific directories, identifies file types, and applies tailored analysis methods:

- For spreadsheet files (CSV, XLSX): extracts row and column counts, headers, and representative sample data using pandas [9].
- For MGF files: counts the number of MS/MS spectra and feature IDs.
- For plain-text files: provides line counts to identify the number of features present.

Output. Produces structured summaries containing file paths, file sizes, and content-specific details for downstream processing.

S3.7 GraphSparqlQAChain

Objective. Generate and execute SPARQL queries aligned with the knowledge graph's schema.

Input. User question, schema information in Turtle format [13], and resolved entity identifiers.

Process — query generation. Uses the LLM with a structured prompt that combines in-context learning [4], chain-of-thought reasoning [14], and one-shot examples to generate a schema-compliant SPARQL SELECT query [15]. The prompt ensures adherence to content clarity, proper prefix usage, schema alignment, and validation requirements.

Process — query refinement. When an initial query yields no results, the tool identifies schema nodes related to the original query and retrieves a similar reference query from a stored FAISS database. Using these inputs, the LLM regenerates a more accurate, schema-compliant query (one refinement iteration).

Process — result processing. Successfully executed query results are saved to a temporary CSV file for downstream analysis. Token usage is monitored to ensure compatibility with the LLM's context window (6,000 token limit); results are truncated if the combined token count of the generated SPARQL query, the user question, and the endpoint output exceeds this threshold, and users are directed to the CSV file for the full output.

Error handling. Removes extraneous elements from generated queries and enforces schema compliance. Manages token limits and optimises the result passed to the LLM.

S3.8 WikidataStructureSearch

Objective. Retrieve compound annotations associated with a taxon (e.g., a plant genus) using its Wikidata entity identifier.

Input. A string representing the Wikidata IRI of a taxon.

Process. Constructs and executes a SPARQL query on the Wikidata public endpoint to retrieve the Wikidata IRIs of chemical compounds annotated to species within the genus of the input taxon. Results are saved to a temporary CSV file.

Output. Path to the CSV file containing compound Wikidata IRIs, or `None` if no results are found.

S3.9 OutputMerger

Objective. Identify overlapping Wikidata compound annotations between results from the ENPKG endpoint and the Wikidata endpoint.

Input. Two file paths to CSV files: one from `GraphSparqlQAChain` (ENPKG endpoint), one from `WikidataStructureSearch` (Wikidata endpoint).

Process. Loads both CSV files, strips and standardises Wikidata IRI formatting, identifies the common IDs between the two datasets, removes duplicates, and saves the final merged list to a temporary CSV file.

Output. Path to a CSV file containing the list of common Wikidata compound IRIs.

S3.10 Interpreter (tool)

Objective. Contextualise query results through appropriate summaries and visualisations.

Input. SPARQL query results (CSV file paths), additional user-uploaded files, and the original user question.

Process.

- *Input processing*: parses agent inputs to extract questions, file paths, and contextual information.
- *Session-based interpretation*: dynamically generates and executes Python code within a secure, custom-built local subprocess sandbox to generate meaningful interpretations; produces JSON code for Plotly [8] graphs on request.

Output. Returns textual summaries and, when visualisations are requested, the file paths of the generated Plotly JSON files.

Output management. Logs processing steps, saves visualisation files to session-specific directories, and provides file paths to users.

S3.11 SpectrumPlotter

Objective. Generate a URL that renders an interactive spectrum plot via the Metabolomics Spectrum Resolver [16] for a given spectrum identifier.

Input. A Universal Spectrum Identifier (USI) [17] value, or a CSV file path containing a column named `usi`.

Process. Once a valid USI is obtained, constructs a URL in the format:

`https://metabolomics-usi.gnps2.org/dashinterface/?usi1=\{usi\}`

Output. The constructed URL where the spectrum plot can be rendered interactively.

S4 SPARQL Query Generation and Refinement — Extended Description

The `GraphSparqlQAChain` tool implements a four-stage pipeline for translating natural-language questions into executable SPARQL queries.

Stage 1 — Initial query generation. The tool combines the user's question, the ENPKG schema in Turtle format [13], and any resolved entity identifiers to generate a SPARQL SELECT query [15]. For example, a question about "bioactive compounds in *Tabernaemontana coffeoides*" would be combined with the Wikidata IRI for *T. coffeoides* (Q15376858) and the relevant schema properties to construct the query. The generation prompt combines in-context learning [4], chain-of-thought reasoning [14], and a one-shot example to guide the LLM towards syntactically correct, schema-compliant output.

Stage 2 — Query execution and result analysis. Generated queries are submitted to the ENPKG SPARQL endpoint, and the results are analysed for completeness and relevance. If a query returns results, execution proceeds to Stage 4. If the query yields no results or raises an error, the tool advances to Stage 3.

Stage 3 — Query refinement. For unsuccessful queries, the tool identifies schema nodes related to the initial query, retrieves one analogous reference query from a FAISS-indexed database of validated example queries, and re-prompts the LLM to regenerate a more accurate, schema-compliant query. This refinement is performed only once per question, guided by concise schema information, step-by-step reasoning, and explicit validation criteria.

An important capability at this stage is the tool's ability to distinguish between errors in query construction and the genuine absence of relevant data in the knowledge graph. For example, if a query about "terpenes in *Tabernaemontana coffeoides*" returns no results, the system first assesses whether the query might be improperly constructed and attempts regeneration. If the regenerated query returns results, this confirms that the initial failure was a construction error. If the regenerated query also fails, this may indicate either persistent construction errors or a genuine absence of the requested data in the graph. This distinction enables targeted refinement strategies and improves overall system reliability.

Stage 4 — Result processing. Successfully executed query results are written to a temporary CSV file. Token usage is monitored to ensure compatibility with the LLM's context window; if the combined token count of the generated query, the user question, and the endpoint output exceeds 6,000 tokens, only the file path is returned to the LLM and users retrieve the complete output directly from the CSV file.

S5 Agent Interaction — Extended Description

Agent interaction is orchestrated through the LangGraph directed state graph (Figure S2), with messages passing along directed edges from one agent to the next. The shared mutable state ensures that each agent operates with the most current and relevant information, enhancing efficiency and accuracy throughout the pipeline.

The structured decision logic is as follows:

1. The **Entry Agent** classifies the incoming query as either a new question (forwarded to the Validator) or a follow-up (forwarded directly to the Supervisor with prior conversation context).

2. The **Validator Agent** verifies schema and data alignment; valid questions proceed to the Supervisor, invalid questions are returned with specific feedback.

3. The **Supervisor Agent** analyses the query for key entities and decides whether to invoke the KG Agent for entity resolution or to forward the question directly to the SPARQL Query Runner Agent.

4. The **KG Agent** resolves entities and returns the identifiers to the Supervisor, which then forwards the enriched query to the SPARQL Query Runner Agent.

5. The **SPARQL Query Runner Agent** generates and executes the SPARQL query, optionally consulting Wikidata, and returns the result to the Supervisor.

6. The **Supervisor** routes the output to the **Interpreter Agent**, which produces the final user-facing response.

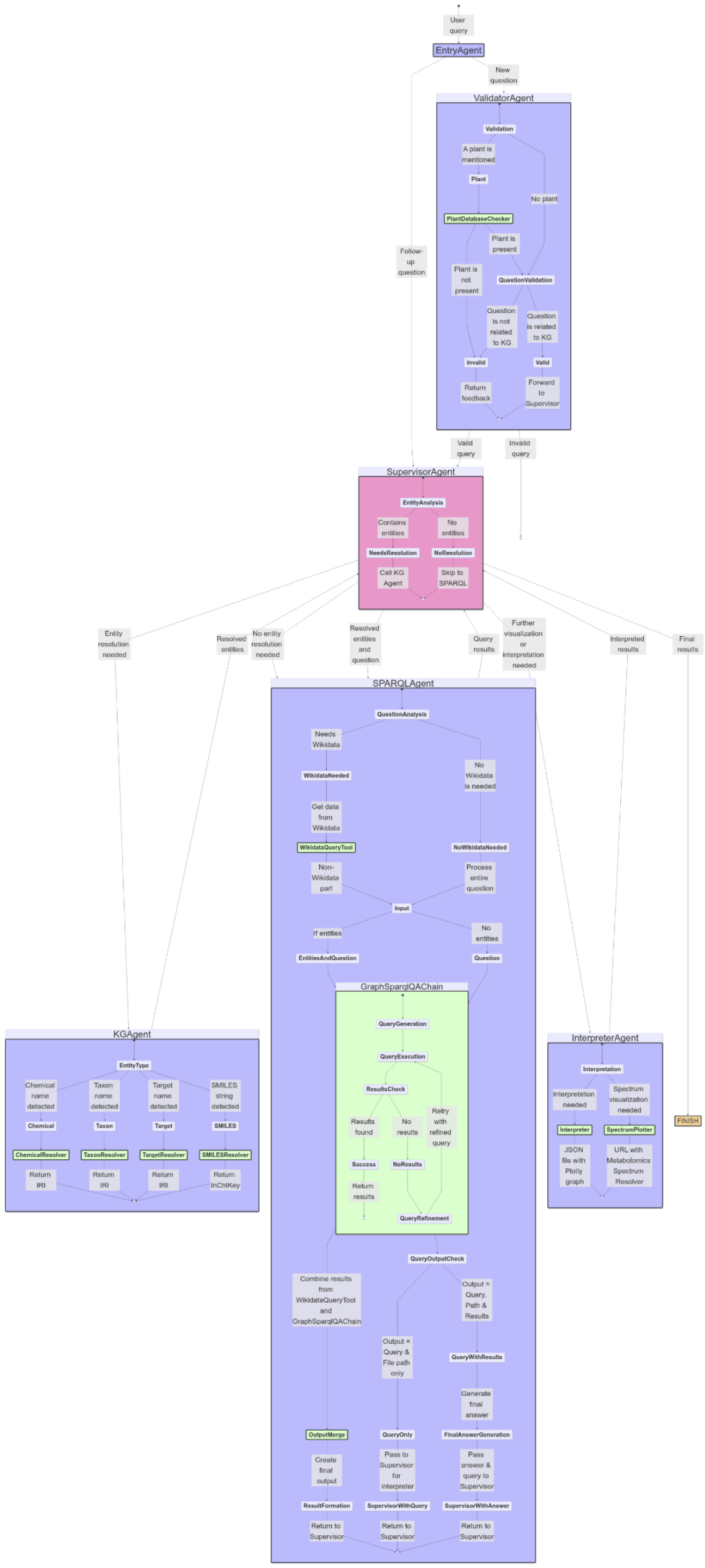

Figure S2: **Agent interaction workflow with decision points.** Sequential decision-making process within MetaboT. A user query initiates the workflow at the Entry Agent, which classifies the question as a new question (routed to the Validator Agent) or a follow-up (directly to the Supervisor Agent). Valid questions proceed to the Supervisor, where entity analysis determines whether to invoke the KG Agent for entity resolution or bypass it for direct SPARQL generation. The SPARQL Query Runner Agent generates and executes the query, then passes its output back to the Supervisor. The output is finally processed by the Interpreter Agent, which produces the user-facing response.

S5.1 LangSmith Monitoring

During development and evaluation, agent interactions were monitored using LangSmith [18] (version 0.3.45), the LangChain platform for developing, testing, and evaluating LLM-based applications. LangSmith provided trace-level visibility into each agent's inputs, outputs, and tool calls, enabling systematic identification of performance bottlenecks and refinement opportunities throughout the development cycle.

S6 Detailed Evaluation Protocol

S6.1 Automated Evaluation Framework

Automated evaluation was conducted using LangSmith's evaluation workflow [18] with LLM-based evaluators implementing the LLM-as-a-Judge paradigm [19]. Three evaluator criteria were applied to each system output:

1. **Correctness** — whether the generated SPARQL query correctly encodes the intent of the natural-language question.
2. **Accuracy** — whether the query returns results that are factually consistent with the reference SPARQL query output.
3. **SPARQL query similarity** — structural and semantic similarity of the generated query to the expert-authored reference query.

LLM-based evaluators may occasionally hallucinate or produce inconsistent judgements [19]; therefore, all automated evaluations were validated through parallel manual review (see Section S6.3).

S6.2 Iterative Evaluation Rounds

Evaluation was performed iteratively across three rounds, with targeted prompt refinements between rounds to address systematically identified failure patterns. Each round processed the full benchmark of 49 questions (50 questions minus 1 excluded due to query-identity artefact; see main text) under identical hardware and network conditions to ensure comparability. Refinements between rounds focused on the system prompts of the agents responsible for the most prevalent error types in the preceding round (primarily the Validator and Supervisor agents).

S6.3 Manual Evaluation

Manual evaluation was performed alongside each automated round by two domain experts who independently reviewed the generated SPARQL queries against the reference queries and the knowledge-graph schema. Discrepancies between manual and automated judgements were resolved by consensus and used to calibrate the LLM evaluators for subsequent rounds.

S6.4 Evaluation Scope

The evaluation assessed all components of the pipeline up to and including SPARQL query generation. The Interpreter Agent, which produces textual summaries and visualisations from query results, was excluded from the quantitative benchmark; its outputs were assessed qualitatively through demonstration queries.

S6.5 Benchmark Dataset Construction

The evaluation dataset of 50 natural-language question–reference SPARQL pairs was constructed as follows:

1. Questions were crafted by domain experts based on the ontology classes and properties present in the ENPKG schema, guided by scientifically meaningful use cases in metabolomics and natural products research.

2. Questions were categorised into three complexity tiers (see Table S2 and main text).

3. Initial SPARQL queries were generated by MetaboT and then manually reviewed and corrected to produce gold-standard reference queries semantically aligned with the full intent of each question.

The complete dataset is available at https://github.com/HolobiomicsLab/MetaboT/tree/main/app/data/evaluation_dataset.csv and archived at https://doi.org/10.5281/zenodo.20748535.

S7 Hardware and Software Requirements

Table S1 summarises the hardware and software configuration on which MetaboT was developed and evaluated.

Table S1: Hardware and software requirements for MetaboT.

**Component**	**Specification**
Hardware	
CPU	Modern processor (Intel Core i7 13th generation or equivalent)
RAM	≥8 GB (recommended)
Operating systems	
	macOS Sonoma 14.5
	Ubuntu 22.04 LTS
	Debian 11
Software	
Python	3.10
LangChain	0.3.27
LangGraph	0.3.34
FAISS	1.8.0
OpenAI Python SDK	2.32.0
pandas	2.2.1
plotly	5.20.0

The system is accessible via a web application interface and can be deployed using the provided Docker configuration or through direct installation following the repository instructions at https://github.com/HolobiomicsLab/MetaboT. Detailed setup information, environment configurations, and dependency management are documented in the project repository.

S8 Full Evaluation Results

Table S2 presents the complete per-system, per-complexity evaluation results including error-type counts for all three configurations assessed on the 49-question benchmark (one question excluded; see main text). Accuracy is reported as the percentage of questions for which the generated SPARQL query was judged correct by the combined automated and manual evaluation.

Table S2: **Full evaluation results across system configurations and query complexity levels.** Numbers in parentheses after error types indicate question counts. T1: Validator misclassification; T2: incorrect SPARQL query; T3: Supervisor failure to invoke the KG Agent; T4: KG Agent tool-selection error. Overall accuracy is computed over all 49 questions. Per-complexity accuracy denominators: Low = 11, Medium = 19, High = 19 questions.

System	Overall	Complexity	Accuracy (%)	Error type(s)
GPT-4o (one-shot)	8.16%	Low	18.18	T2
		Medium	10.53	T2
		High	0.00	T2
MetaboT GPT-4o mini	12.24%	Low	0.00	T1 (7), T2 (4)
		Medium	15.79	T1 (7), T2 (9)
		High	15.79	T1 (6), T2 (8), T3 (1), T4 (1)
MetaboT GPT-4o	83.67%	Low	81.82	T1 (2)
		Medium	89.47	T2 (1), T3 (1)
		High	78.95	T2 (4)

S9 Worked-Example Output and Web Interface

This section provides the MetaboT-generated plot for the bioactive-metabolite worked example discussed in the main text (Fig. 3) and a step-by-step guide to the web interface.

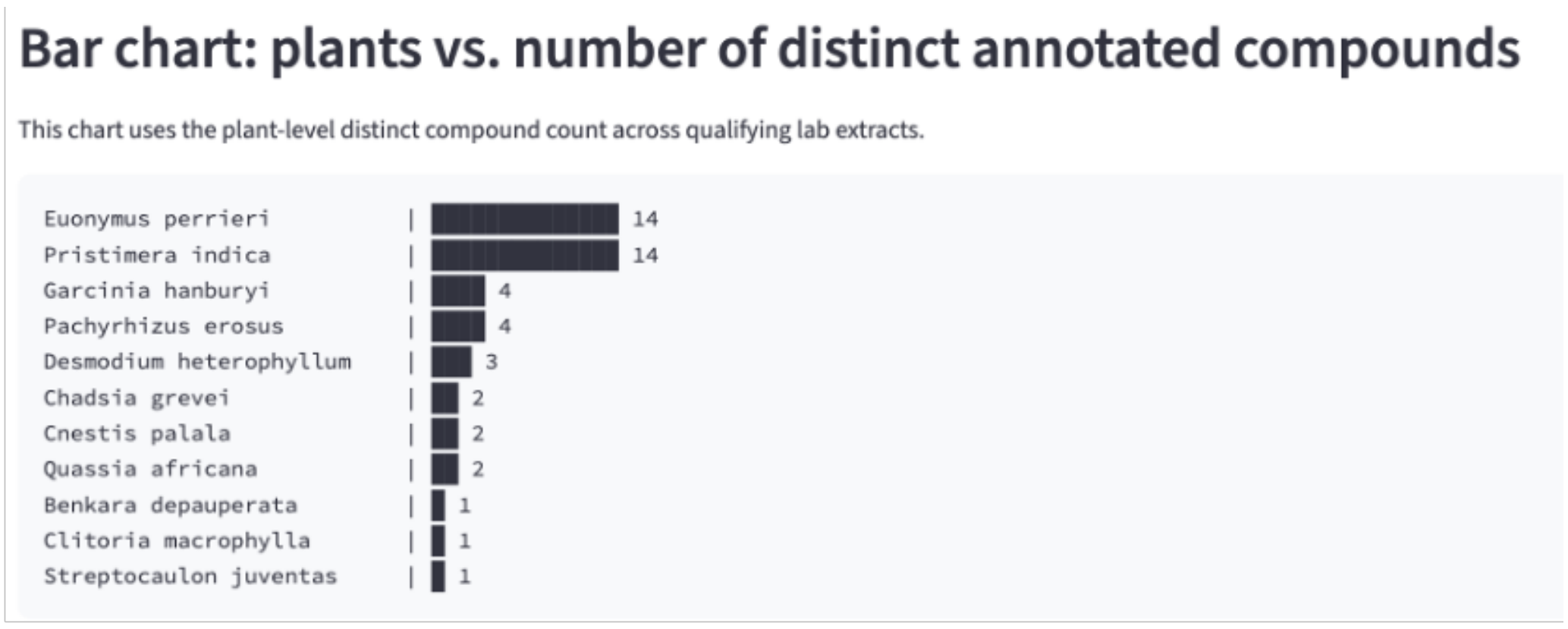

Figure S3: Plot generated by MetaboT for the Fig. 3 worked example, returned when the query is re-issued with a request to visualise the result (11 plants qualify).

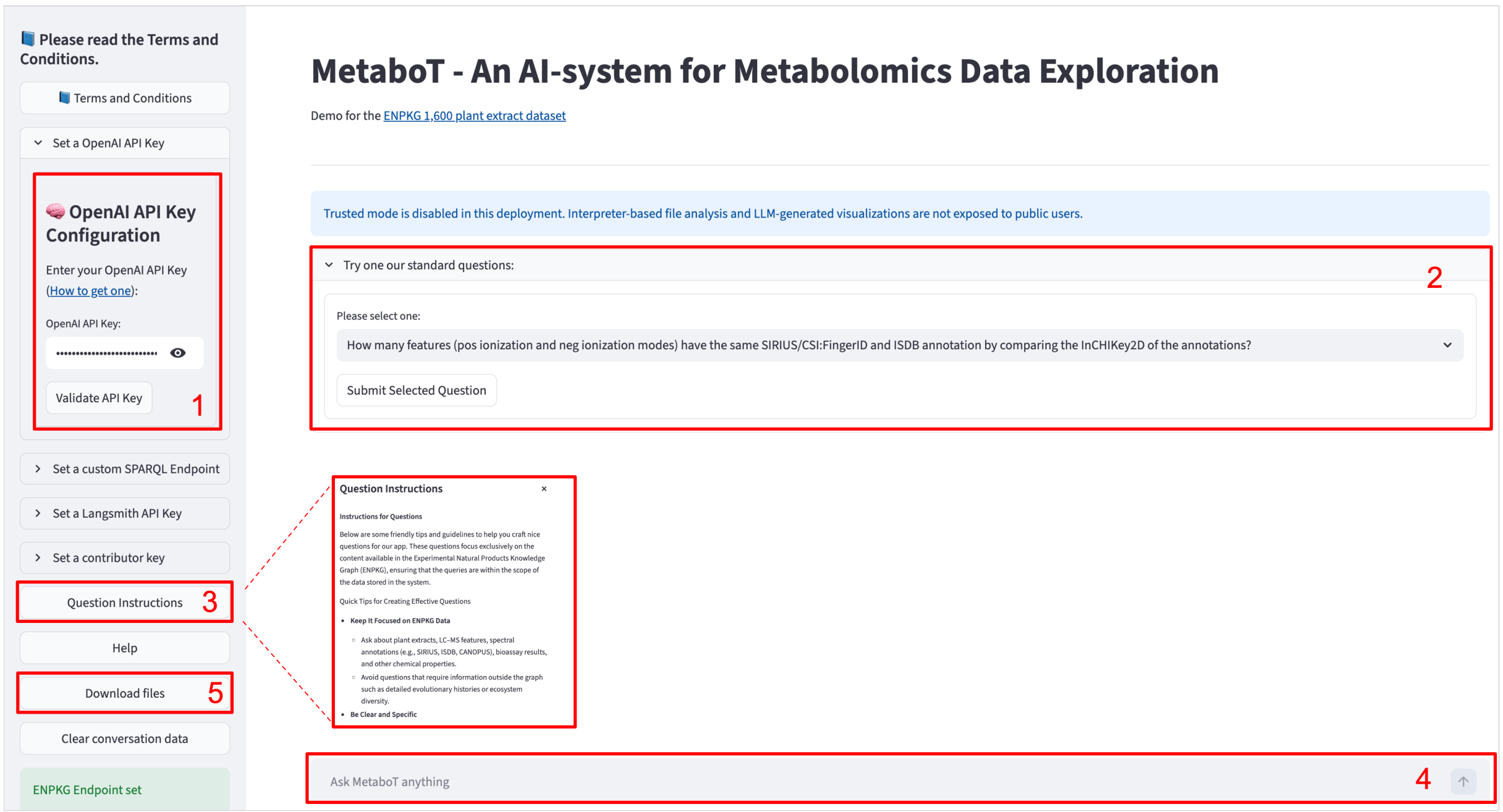

Figure S4: Step-by-step guide to the MetaboT web interface. (1) Enter your OpenAI API key and validate it; (2) try one of the standard questions bundled in the web application; (3) open the Question Instructions panel to read the guidance for crafting custom questions; (4) submit your question; (5) download the results.

References

[1] LangChain: LangChain. https://www.langchain.com (2024)

[2] LangChain: LangGraph. https://www.langchain.com/langgraph (2024)

[3] Topsakal, O., Akinci, T.C.: Creating large language model applications utilising LangChain: a primer on developing LLM apps fast. In: Proceedings of the International Conference on Applied Engineering and Natural Sciences, vol. 1, pp. 1050–1056 (2023). https://doi.org/10.59287/icaens.1127

[4] Dong, Q., Li, L., Dai, D., Zheng, C., Ma, J., Li, R., Xia, H., Xu, J., Wu, Z., Chang, B., Sun, X., Li, L., Sui, Z.: A survey on in-context learning. In: Al-Onaizan, Y., Bansal, M., Chen, Y.-N. (eds.) Proceedings of the 2024 Conference on Empirical Methods in Natural Language Processing, pp. 1107–1128. Association for Computational Linguistics, Miami, Florida, USA (2024). https://doi.org/10.18653/v1/2024.emnlp-main.64 . https://aclanthology.org/2024.emnlp-main.64/

[5] Wang, M., Carver, J.J., Phelan, V.V., Sanchez, L.M., Garg, N., Peng, Y., Nguyen, D.D., Watrous, J., Kapono, C.A., Luzzatto-Knaan, T., Porto, C., Bouslimani, A., Melnik, A.V., Meehan, M.J., Liu, W.-T., Crüsemann, M., Boudreau, P.D., Esquenazi, E., Sandoval-Calderón, M., Kersten, R.D., Pace, L.A., Quinn, R.A., Duncan, K.R., Hsu, C.-C., Floros, D.J., Gavilan, R.G., Kleigrewe, K., Northen, T., Dutton, R.J., Parrot, D., Carlson, E.E., Aigle, B., Michelsen, C.F., Jelsbak, L., Sohlenkamp, C., Pevzner, P., Edlund, A., McLean, J., Piel, J., Murphy, B.T., Gerwick, L., Liaw, C.-C., Yang, Y.-L., Humpf, H.-U., Maansson, M., Keyzers, R.A., Sims, A.C., Johnson, A.R., Sidebottom, A.M., Sedio, B.E., Klitgaard, A., Larson, C.B., P., C.A.B., Torres-Mendoza, D., Gonzalez, D.J., Silva, D.B., Marques, L.M., Demarque, D.P., Pociute, E., O'Neill, E.C., Briand, E., Helfrich, E.J.N., Granatosky, E.A., Glukhov, E., Ryffel, F., Houson, H., Mohimani, H., Kharbush, J.J., Zeng, Y., Vorholt, J.A., Kurita, K.L., Charusanti, P., McPhail, K.L., Nielsen, K.F., Vuong, L., Elfeki, M., Traxler, M.F., Engene, N., Koyama, N., Vining, O.B., Baric, R., Silva, R.R., Mascuch, S.J., Tomasi, S., Jenkins, S., Macherla, V., Hoffman, T., Agarwal, V., Williams, P.G., Dai, J., Neupane, R., Gurr, J., Rodríguez, A.M.C., Lamsa, A., Zhang, C., Dorrestein, K., Duggan, B.M., Almaliti, J., Allard, P.-M., Phapale, P., Nothias, L.-F., Alexandrov, T., Litaudon, M., Wolfender, J.-L., Kyle, J.E., Metz, T.O., Peryea, T., Nguyen, D.-T., VanLeer, D., Shinn, P., Jadhav, A., Müller, R., Waters, K.M., Shi, W., Liu, X., Zhang, L., Knight, R., Jensen, P.R., Palsson, B.Ø., Pogliano, K., Linington, R.G., Gutiérrez, M., Lopes, N.P., Gerwick, W.H., Moore, B.S., Dorrestein, P.C., Bandeira, N.: Sharing and community curation of mass spectrometry data with Global Natural Products Social Molecular Networking. Nat Biotechnol **34**, 828–837 (2016) https://doi.org/10.1038/nbt.3597

[6] Zdrazil, B., Felix, E., Hunter, F., Manners, E.J., Blackshaw, J., Corbett, S., Veij, M., Ioannidis, H., Lopez, D.M., Mosquera, J.F., Magariños, M.P., Bosc, N., Arcila, R., Kizilören, T., Gaulton, A., Bento, A.P., Adasme, M.F., Monecke, P., Landrum, G.A., Leach, A.R.: The ChEMBL database in 2023: a drug discovery platform spanning multiple bioactivity data types and time periods. Nucleic Acids Res **52**, 1180–1192 (2024) https://doi.org/10.1093/nar/gkad1004

[7] Waagmeester, A., Stupp, G., Burgstaller-Muehlbacher, S., Good, B.M., Griffith, M., Griffith, O.L., Hanspers, K., Hermjakob, H., Hudson, T.S., Hybiske, K., Keating, S.M., Manske, M., Mayers, M., Mietchen, D., Mitraka, E., Pico, A.R., Putman, T., Riutta, A., Queralt-Rosinach, N., Schriml, L.M., Shafee, T., Slenter, D., Stephan, R., Thornton, K., Tsueng, G., Tu, R., Ul-Hasan, S., Willighagen, E., Wu, C., Su, A.I.: Science forum: Wikidata as a knowledge graph for the life sciences. eLife **9**, 52614 (2020) https://doi.org/10.7554/eLife.52614

[8] Plotly Technologies Inc.: Plotly Python graphing library. https://plotly.com/python/ (2015)

[9] Johnson, J., Douze, M., Jégou, H.: Billion-scale similarity search with GPUs. IEEE Trans Big Data **7**, 535–547 (2021) https://doi.org/10.1109/TBDATA.2019.2921572

[10] OpenAI: text-embedding-ada-002 and subsequent embedding models. https://platform.openai.com/docs/guides/embeddings (2024)

[11] Weininger, D.: SMILES, a chemical language and information system. 1. Introduction to methodology and encoding rules. J Chem Inf Comput Sci **28**, 31–36 (1988) https://doi.org/10.1021/ci00057a005

[12] Heller, S., McNaught, A., Stein, S., Tchekhovskoi, D., Pletnev, I.: InChI – the worldwide chemical structure identifier standard. J Cheminform **5**, 7 (2013) https://doi.org/10.1186/1758-2946-5-7

[13] Beckett, D., Berners-Lee, T., Prud'hommeaux, E., Carothers, G.: RDF 1.1 Turtle: terse RDF triple language. W3C Recommendation (2014). https://www.w3.org/TR/turtle/

[14] Wei, J., *et al.*: Chain-of-thought prompting elicits reasoning in large language models. In: Advances in Neural Information Processing Systems, vol. 35, pp. 24824–24837 (2022). https://arxiv.org/abs/2201.11903

[15] Harris, S., Seaborne, A.: SPARQL 1.1 Query Language. W3C Recommendation (2013). https://www.w3.org/TR/sparql11-query/

[16] Bittremieux, W., Chen, C., Dorrestein, P.C., Schymanski, E.L., Schulze, T., Neumann, S., Meier, R., Rogers, S., Wang, M.: Universal MS/MS visualization and retrieval with the Metabolomics Spectrum Resolver web service. bioRxiv (2020) https://doi.org/10.1101/2020.05.09.086066

[17] HUPO Proteomics Standards Initiative: Universal Spectrum Identifier. https://www.psidev.info/usi (2023)

[18] LangChain: LangSmith. https://www.langchain.com/langsmith (2024)

[19] Gu, J., et al.: A survey on LLM-as-a-judge. arXiv (2025). https://doi.org/10.48550/arXiv.2411.15594
